# TLR2-induced astrocyte MMP9 activation compromises the blood brain barrier and exacerbates intracerebral hemorrhage in animal models

**DOI:** 10.1186/s13041-015-0116-z

**Published:** 2015-04-10

**Authors:** Hyunjung Min, Jinpyo Hong, Ik-Hyun Cho, Yong Ho Jang, Hyunkyoung Lee, Dongwoon Kim, Seong-Woon Yu, Soojin Lee, Sung Joong Lee

**Affiliations:** Department of Neuroscience and Physiology, and Dental Research Institute, School of Dentistry, Seoul National University, Seoul, 110-749 South Korea; Department of Convergence Medical Science, College of Oriental Medicine, Kyung Hee University, Seoul, 130-701 South Korea; Department of Anatomy, Brain Research Institute, School of Medicine, Chungnam National University, Daejeon, 305-764 South Korea; Department of Brain Science, Daegu Gyeongbuk Institute of Science and Technology, Daegu, 711-873 Republic of Korea; Department of Microbiology and Molecular Biology, Chungnam National University, Daejeon, 305-764 South Korea

**Keywords:** Toll-like receptor, Stroke, Neuroinflammation, Neutrophil, Matrix metalloproteinase-9

## Abstract

**Background:**

The innate immune response plays an important role in the pathogenesis of intracerebral hemorrhage (ICH). Recent studies have shown that Toll-like receptor 2 (TLR2) is involved in the innate immune response in various neurological diseases, yet neither its role in ICH nor the mechanisms by which it functions have yet been elucidated. We examined these in this study using a collagenase-induced mouse ICH model with TLR2 knock-out (KO) mice.

**Results:**

TLR2 expression was upregulated in the ipsilateral hemorrhagic tissues of the collagenase-injected mice. Brain injury volume and neurological deficits following ICH were reduced in TLR2 KO mice compared to wild-type (WT) control mice. Heterologous blood-transfer experiments show that TLR2 signaling in brain-resident cells, but not leukocytes, contributes to the injury. In our study to elucidate underlying mechanisms, we found that damage to blood–brain barrier (BBB) integrity following ICH was attenuated in TLR2 KO mice compared to WT mice, which may be due to reduced matrix metalloproteinase-9 (MMP9) activation in astrocytes. The reduced BBB damage accompanies decreased neutrophil infiltration and proinflammatory gene expression in the injured brain parenchyma, which may account for the attenuated brain damage in TLR2 KO mice after ICH.

**Conclusions:**

TLR2 plays a detrimental role in ICH-induced brain damage by activating MMP9 in astrocytes, compromising BBB, and enhancing neutrophils infiltration and proinflammatory gene expression.

## Background

Intracerebral hemorrhage (ICH) is one of the major types of stroke and accounts for 15% to 20% of all stroke cases. In ICH, the initial insult by the mechanical force of the expanding hematoma and plasma proteins is usually followed by secondary damage, which involves inflammatory responses in the perihematomal region [1,2]. The inflammatory responses accompany activation of brain-resident glial cells including microglia and astrocytes, disruption of blood–brain barrier (BBB) and subsequent edema, leukocyte recruitment to the injury site, and induction of proinflammatory and potentially neurotoxic mediators [3-7]. The concerted effects of these inflammatory events result in massive neuronal death leading to further sustained and aggravated neurological damage subsequent to the stroke. It has been suggested that hematoma release and its degradation products may activate inflammatory responses at the perihematomal region following ICH [8]. In addition, activation of matrix metalloproteinases (MMPs) has been implicated in the disruption of the BBB and perihematomal edema formation [9-11]. However, the mechanisms underlying the MMP activation and inflammatory responses following ICH have not been clearly elucidated.

Toll-like receptor 2 (TLR2) is a type I transmembrane receptor that recognizes lipoteichoic acid (LTA) and peptidoglycan of bacterial cell walls, and thereupon triggers inflammatory signals in innate immune cells. Recent studies have shown that TLR2 functions as a receptor not only for pathogen-associated molecular patterns, but also for endogenous molecules released from damaged tissues or cells, such as heat shock proteins (HSPs) [12-14], hyaluronan [15], soluble CD14 [16] and high mobility group box-1 (HMGB-1) [17,18]. Studies suggest that TLR2 activation by these endogenous “danger signals” is involved in the development of neuroinflammatory responses in various neurological disorders [19,20]. For example, in traumatic brain injury, activation of microglia and astrocytes in response to injury is dependent on TLR2 signaling, which induces subsequent proinflammatory gene expression around the lesion site of a stab wound [21]. Likewise, TLR2 is required for nerve injury-induced spinal cord microglia activation and proinflammatory gene expression in the spinal cord [22]. However, the function of TLR2 in ischemic brain injury remains controversial. TLR2-deficient mice displayed decreased brain injury and leukocyte infiltration compared to wild-type (WT) mice, indicating a detrimental and proinflammatory role of TLR2 in ischemic stroke [23,24]. However, in another study, TLR2 knock-out (KO) mice showed higher mortality, decreased neurological function, and increased brain infarct size [25], indicating a neuroprotective role for TLR2. In line with a neuroprotective TLR2 role, upon spinal cord injury (SCI), locomotor deficit is aggravated and recovery from injury is impaired in TLR2-deficient mice [26]. Thus, in spinal cord injury, TLR2-mediated innate immune responses seem to facilitate recovery after injury. Despite these studies implicating TLR2 in ischemic stroke, its role in ICH has not been elucidated thus far. In this study, we investigated the role of TLR2 in secondary inflammatory brain damage following ICH using TLR2 KO mice in a collagenase-induced ICH model.

## Results

### ICH-induced brain damage and neurologic deficits are reduced in TLR2 KO mice

**Figure 1:**
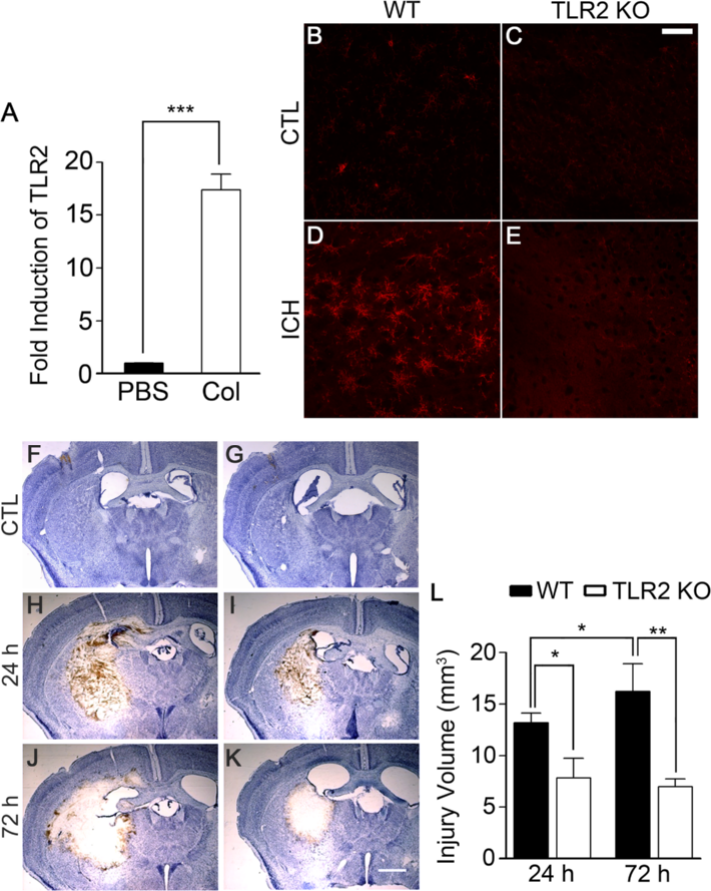
**ICH-induced brain injury volume is reduced in TLR2 KO mice.** WT and TLR2 KO mice were treated with ICH by collagenase injection (0.075 U in 0.5 μl PBS). **(A)** At 6 h following PBS or collagenase injection, total RNA was prepared from ipsilateral hemorrhagic tissue in WT mice (n = 3) and used for quantitative real-time RT-PCR to measure TLR2 mRNA level. Data are expressed as mean ± SEM (*** *p* < 0.001, vs. PBS-injected WT mice). **(B-E)** At 24 h after collagenase injection, the brains were sectioned and stained with TLR2 antibody. Scale bar: 50 μm. WT and TLR2 KO mice (n = 5) were injected with either PBS **(F and** **G)** or collagenase **(H-K)**, and the brains were sectioned and stained with cresyl violet at 24 or 72 h following injection. Representative pictures are shown. Scale bar: 200 μm. **(L)** Injury volume (mm^3^) was calculated by multiplying section thickness and injured hemorrhagic areas. Data are expressed as mean ± SEM (** *p* < 0.01, * *p* < 0.05, vs. collagenase-injected WT mice, n = 5).

To test the involvement of TLR2 in ICH-type stroke, we adopted a collagenase-induced ICH model, which is a well-characterized animal model for ICH [27]. To begin the study, we measured TLR2 expression in ICH-injured mouse brain. After collagenase injection, TLR2 mRNA (Figure 1A) and protein (Figure 1B-E) expression were upregulated in WT mouse. To investigate the role of TLR2 in ICH, we compared collagenase-induced ICH injuries in WT and TLR2 KO mice brains. At 24 h post-collagenase injection, severe hemorrhagic lesions were detected in the stratum around the injection site of the cresyl violet-stained brain sections from the WT mice (Figure 1H). The lesion areas had slightly further increased by 72 h post-injection (Figure 1J). In the TLR2 KO mice, the lesion areas were markedly smaller than in the WT mice (Figure 1I and K). In both the WT and TLR2 KO mice, no detectable hemorrhagic lesions were observed in sham-operated mice (Figure 1F and G). Quantification of injury volume confirmed that the hemorrhagic volume was smaller in TLR2 KO mice than in WT mice both at 24 h (10.5 vs. 6.3 mm^3^) and 72 h (13.0 vs. 5.6 mm^3^) post-injection (Figure 1L).

**Figure 2:**
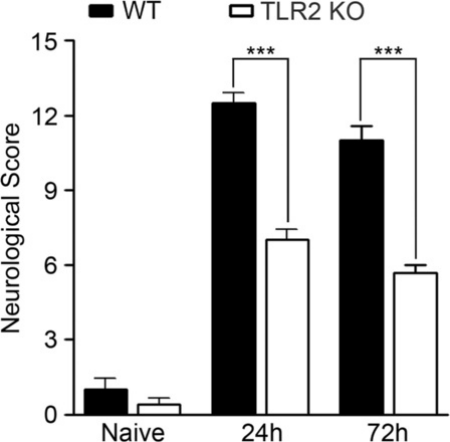
**Neurological deficit after ICH is attenuated in TLR2 KO mice.** At 24 and 72 h following collagenase injection, neurological deficits in WT (n = 5) and TLR2 KO (n = 5) mice were evaluated using a 28-point neurological scoring system. Neurological scores of WT and TLR2 KO mice before collagenase injection are presented as control data (naïve). Data are presented as mean ± SEM (*** *p* < 0.001, vs. collagenase-injected WT mice).

ICH is usually accompanied by characteristic behavioral deficits. To assess whether the reduced hemorrhagic volumes in TLR2 KO mice manifested as reduced neurological deficits, neurobehavioral tests were carried out on the WT and TLR2 KO mice using a 28-point neurological scoring system [27]. At 24 and 72 h post-collagenase injection, neurological scores were significantly lower in TLR2 KO mice than in WT mice (12.5 vs 7.0 at 24 h, 11.0 vs 5.7 at 72 h) (Figure 2). These data demonstrate that both post-hemorrhagic brain damage and neurological deficits are reduced in the TLR2 KO mice.

**Figure 3:**
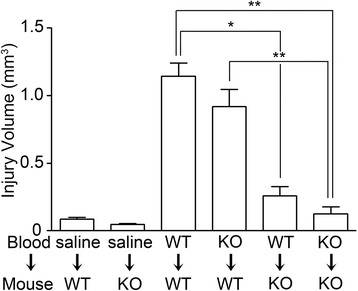
**TLR2 expressed in brain parenchyma contributes to ICH-induced brain damage.** WT and TLR2 KO mice (n = 5) were injected with either WT or TLR KO mouse blood (20 μl) in the striatum: WT blood injection to WT mouse (WT → WT) or to TLR2 KO mouse (WT → KO). TLR2 KO blood injection to WT mouse (KO → WT) or to TLR2 KO mouse (KO → KO). Saline (20 μl) was injected to WT mouse (saline → WT) or TLR2 KO mouse (saline → KO) as control. After 72 h, brain sections were prepared for cresyl violet staining, and hemorrhagic injury volume was calculated. Data are presented as mean ± SEM (** *p* < 0.01, * *p* < 0.05).

Besides the collagenase-induced ICH model, ICH can be mimicked by direct blood injection into the brain parenchyma. This is another well-established ICH animal model [28]. To test whether TLR2 is involved in this ICH model as well, we measured the brain injury volume in WT and TLR2 KO mice after autologous blood injection (Figure 3). When compared to the WT mice, the lesion volume of autologous blood-injected TLR2 KO mice was reduced more than 85% (1.14 mm^3^ vs. 0.13 mm^3^), demonstrating that TLR2 contributes to ICH damage in the blood-injection model as well. The injury volume was also significantly reduced when the WT blood was injected into the TLR2 KO mice, but not when TLR2 KO blood was injected into WT mice. These data suggest that the effects observed in TLR2 KO mice were not due to TLR2 deficiency in the brain-injected blood cells, rather the effects were due to TLR2 deficiency in the resident brain cells.

### TLR2 signaling contributes to BBB damage after ICH

**Figure 4:**
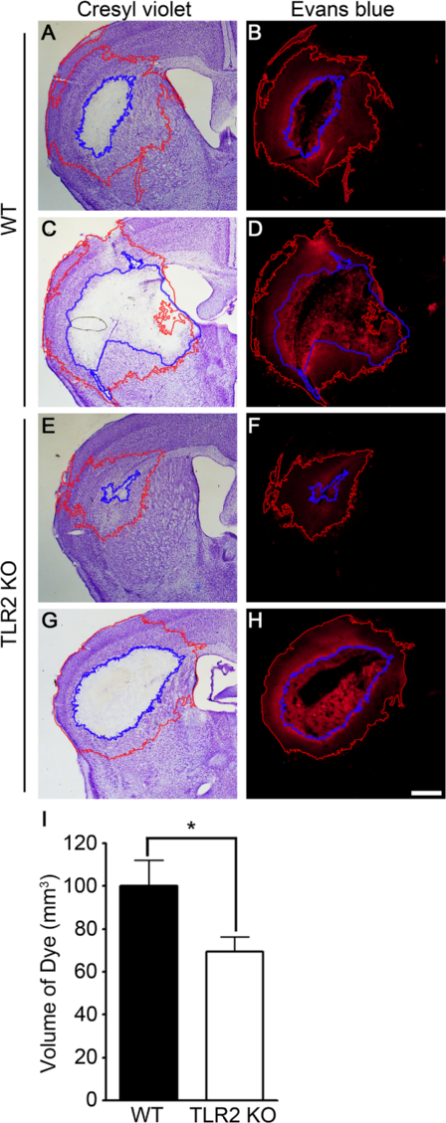
**TLR2 signaling contributes to collagenase-induced BBB damage. (A-H)** WT and TLR2 KO mice (n = 6) were injected with Evans blue dye 2 days after ICH. ICH-injured brains were sectioned at 3 days, and evaluated with either cresyl violet staining **(A,** **C,** **E, and** **G)** or Evans blue staining under light microscope **(B,** **D,** **F, and** **H)**. Serial sections of two different brain regions were stained: AP, −0.85 **(C,** **D,** **G, and** **H)** and AP, −0.45 **(A,** **B,** **E, and** **F)**. Representative pictures are shown. The Evans blue-stained brain volume was calculated and presented in a graph **(I)**. Data are expressed as mean ± SEM (* *p* < 0.05, vs. collagenase-injected WT mice).

Collagenase injected into the brain parenchyma supposedly induces hemorrhage by degrading extracellular matrix and disrupting the BBB. To test if TLR2 is involved in BBB damage due to collagenase injection, we intravenously injected Evans blue dye into the ICH-damaged WT and TLR2 KO mice and assessed BBB integrity by measuring dye leakage into the brain parenchyma. In the ICH-damaged mouse brain, Evans blue dye leakage was detected around the lesion area boundary. In particular, TLR2 KO mice had smaller dye-stained areas than WT mice (Figure 4A-H). Dye-stained volume in the TLR2 KO mice brain was only 69% of that in WT mice (Figure 4I). In both the WT and TLR2 KO mice, Evans blue dye leakage was not detected in sham-control mice (data not shown). Taken together, these data indicate that there is less severe BBB damage in TLR2 KO mice upon collagenase injection.

### TLR2 stimulation induces MMP9 activation in brain astrocytes

**Figure 5:**
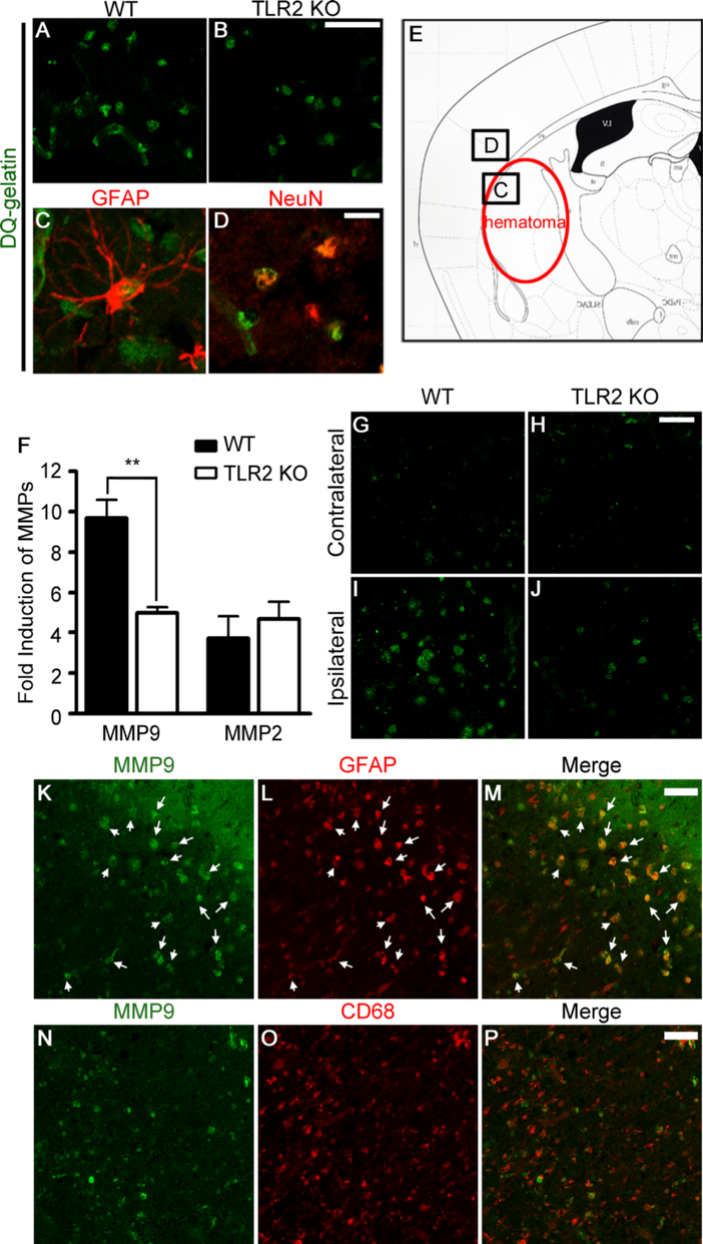
**ICH-induced gelatinase activation is compromised in TLR2 KO mice. (A-B)** At 24 h post-ICH, mice were sacrificed and cryosections were incubated in fluorescein-conjugated DQ gelatin for 2 h. Fluorescence due to gelatinase activity in the perihematoma region was visualized under fluorescent microscope. Scale bars: 50 μm. **(C-D)** Following *in situ* zymography, sections from WT mice were immunostained with anti-GFAP **(C)** or NeuN **(D)** antibodies. The locations of the gelatinase activity shown in the panels **C** and **D** are denoted in panel **E**. Scale bars: 25 μm. **(F)** At 6 h post-collagenase injection, total RNA was isolated from ipsilateral hemorrhagic tissue of WT and TLR2 KO mice (n = 4) and used to measure MMP2 and MMP9 mRNA levels. Data are presented as mean ± SEM (** *p* < 0.01, vs. collagenase-injected WT mice). **(G-J)** Brain sections of WT or TLR2 KO ICH mice (n = 3) were immunostained with anti-MMP9 antibody 24 h after collagenase injection. **(K-P)** ICH injured brain sections obtained from WT or TLR2 KO mice were co-stained with anti-MMP9 and anti-GFAP (K-M) or anti-CD68 **(N-P)** antibodies to determine the cellular source of MMP9. Scale bars: 50 μm.

Given that MMP9 is a key factor responsible for BBB damage [10,29], we tested if TLR2-mediated signaling affects gelatinase activity in ICH brains using *in situ* zymography. Twelve hours post-injection, gelatinolytic activity was observed in the ipsilateral perihematomal regions of the WT mice (Figure 5A) indicating significant activation of MMPs after ICH. Of note, ICH-induced gelatinolytic activity was much lower in TLR2 KO mice brains than in those of WT mice (Figure 5B). To determine the cellular sources of gelatinase activity, brain sections were immunostained with cell type-specific antibodies. In the perihematomal region of ICH-induced WT brains, gelatinase activity was detected in GFAP-immunoreactive (IR) astrocytes (Figure 5C). Outside the perihematomal region (Figure 5E), gelatinase activity was localized to NeuN-IR neurons (Figure 5D). However, no conspicuous gelatinase activity was detected in CD11b-IR microglia or CNPase-IR oligodendrocytes (data not shown). Since both MMP2 and −9 confer gelatinase activity, we tested the regulation of MMP2 and −9 gene expression. Following an ICH, MMP9 transcript increased up to 10-fold in the WT mice brains, whereas the induction level decreased by 48% in the TLR2 KO mice (Figure 5F). Meanwhile, the ICH-induced MMP2 transcript level did not differ between WT and TLR2 KO mice (Figure 5F). Comparably, ICH induced MMP9 protein expression in WT mice brains, which was not as significant in TLR2 KO mice brains (Figure 5G-J). MMP9 expression was primarily detected in GFAP-IR astrocytes (Figure 5K-M, arrows) and not in CD68-IR macrophages/microglia (Figure 5N-P); this suggests that ICH-induced MMP9 expression in astrocytes may have been responsible for the differential gelatinolytic activity observed in brains of WT mice versus TLR2 KO mice.

**Figure 6:**
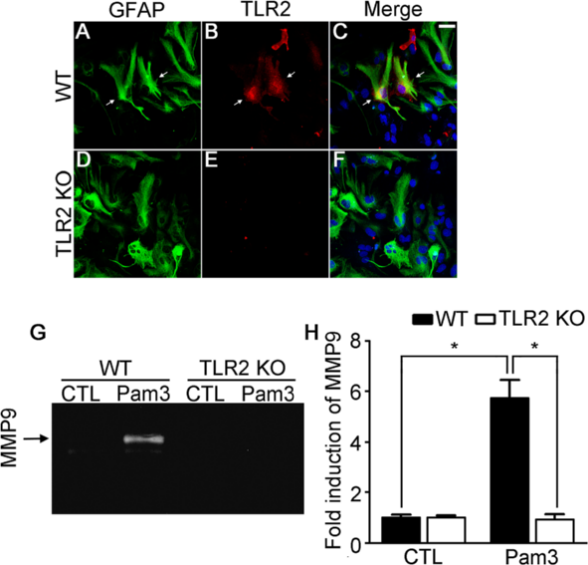
**TLR2 signaling induces MMP9 activity in astrocytes. (A-F)** Primary cerebral glia cultures prepared from WT and TLR2 KO mice were immunostained with anti-GFAP **(A and** **D)** and TLR2 **(B and** **E)** antibodies. Merged pictures are shown **(C and** **F)**. **(G)** Primary astrocytes were prepared from WT and TLR2 KO mice and stimulated with Pam3 (2 μg/ml). After 24 h, medium was collected and MMP9 activity was determined by gel zymography. **(H)** MMP9 transcript was determined by real-time RT-PCR in primary astrocytes at 6 h after Pam3 stimulation. Data are presented as mean ± SEM (* *p* < 0.01).

Since astrocytes are key components of the intact BBB, it is conceivable that TLR2 activation on this cell type may directly induce MMP9 activity and thereby compromise the BBB in the perihematomal region. To address such a possibility, we first tested TLR2 expression on astrocytes. In primary cultured mixed glia from WT mouse cerebra, TLR2-IR was detected in a subpopulation of astrocytes (Figure 6A-C, arrows). TLR2-IR was not detected in TLR2 KO astrocytes, demonstrating the specificity of the TLR2 antibody (Figure 6E). Activation of TLR2 on astrocytes by Pam3, a synthetic TLR2 agonist, induced MMP9 enzymatic activity in the conditioned media, as measured by gel zymography (Figure 6G), whereas the Pam3-induced MMP9 activity was abrogated in astrocytes from TLR2 KO mice (Figure 6G), demonstrating the TLR2-dependent activation of MMP9. We then tested MMP9 expression in primary astrocytes. TLR2 stimulation induced MMP9 mRNA expression more than 5.8-fold in WT astrocytes, but it was almost completely blocked in TLR2 KO astrocytes (Figure 6H). These data indicate that stimulation of TLR2 on astrocytes induces MMP9 expression as well as activity.

### Neutrophil infiltration following ICH is attenuated in TLR2 KO mice

**Figure 7:**
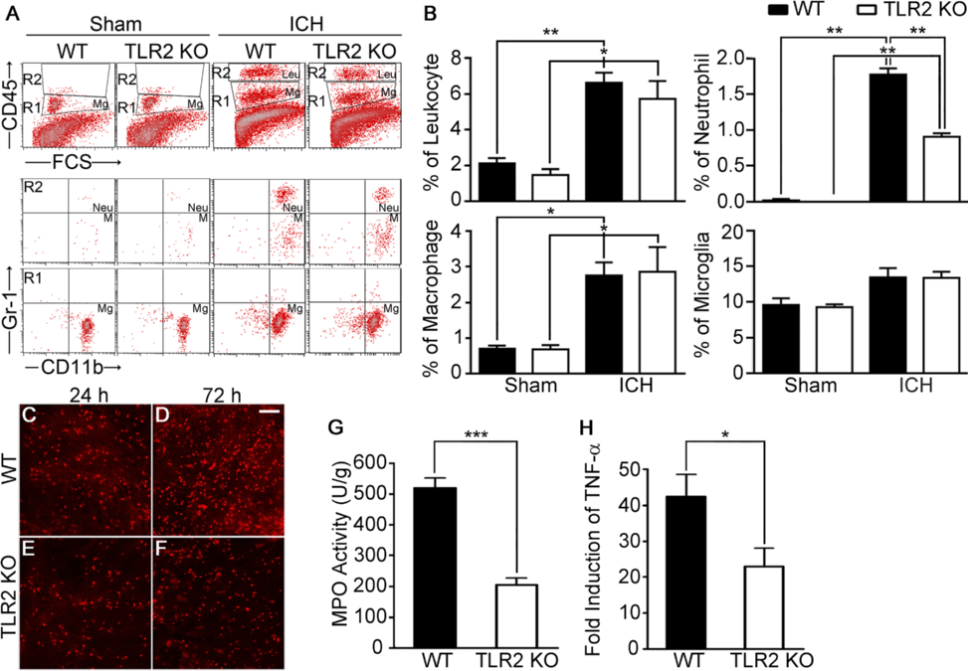
**Neutrophil infiltration following ICH is attenuated in TLR2 KO mice. (A-B)** WT and TLR2 KO mice (n = 3) were subjected to collagenase-induced ICH. After 24 h, brains were dissociated, and the percentages of leukocytes (Leu; CD45^hi^, CD11b^+^), macrophages (M; CD45^hi^, CD11b^+^, Gr-1^low^), neutrophils (Neu; CD45^hi^, CD11b^+^, Gr-1^hi^), and microglia (Mg; CD45^int^, CD11b^+^) present in ICH-injured brain were determined by flow cytometry. Data are presented as mean ± SEM (** *p* < 0.01, * *p* < 0.05). **(C-F)** At 24 h (C and E) and 72 h (D and F) following collagenase injection, brain sections of WT and TLR2 KO mice (n = 5) were immunostained with anti-Gr-1 antibodies to detect neutrophil infiltration. Scale bars: 50 μm. **(G)** MPO activity (U/g) was measured in the ipsilateral brain hemisphere of WT and TLR2 KO mice 24 h post-ICH. Data are expressed as mean ± SEM (*** *p* < 0.001, vs. collagenase-injected WT mice, n = 5). **(H)** Total RNA was isolated from ipsilateral hemorrhagic tissue (AP, 0.0 to −2.0 mm) 6 h post-ICH and used for quantitative real-time RT-PCR to measure TNF-α mRNA levels. Data are expressed as mean ± SEM (* *p* < 0.05, ** *p* < 0.01 vs. collagenase-injected WT mice, n = 3).

Compromise of the BBB often results in immune cell infiltration into brain parenchyma, which is implicated in brain damage after collagenase-induced ICH [30]. To test if TLR2-mediated BBB compromise affects immune cell infiltration, we used flow cytometry to analyze brain lesion-infiltrating immune cells (CD11b^+^/CD45^hi^) and brain-resident microglia (CD11b^+^/CD45^int^) (Figure 7A). The percentage of leukocytes (CD11b^+^/CD45^hi^) in brain parenchyma 72 h after ICH increased from 2.1% to 6.6% (Figure 7B). Of these 6.6% of leukocytes recruited to the brain upon ICH, macrophages (CD11b^+^/CD45^hi^/Gr1^low^) accounted for 2.6%, and neutrophils (CD11b^+^/CD45^hi^/Gr1^hi^) 1.7%, while the number of microglia (CD11b^+^/CD45^int^) was not greatly altered (Figure 7B). In the ICH-induced TLR2 KO brain parenchyma, comparable numbers of macrophages and microglia were detected by flow cytometry (Figure 7A and B). However, the percentage of brain-infiltrating neutrophils in the TLR2 KO mice was reduced by more than 50% compared to WT mice (Figure 7B). To further confirm the putative correlation between BBB damage and neutrophil infiltration in WT and TLR2 KO mice, we measured tissue-infiltrating neutrophils by immunohistochemistry using antibodies against Gr-1. Gr-1-IR neutrophils were detected at 24 h post-injection, and the number was further increased at 72 h (Figure 7C and D). Compared to in WT mice, Gr-1-IR neutrophils were markedly reduced in the TLR2 KO mouse brain (Figure 7E and F). In both WT and TLR2 KO mice, Gr-1-IR cells were rarely detected after sham-operation (data not shown). Brain-infiltrating neutrophils have been reported to inflict brain damage [31,32] by producing reactive oxygen species (ROS) via myeloid peroxidase (MPO) enzyme activity and TNF-α production [33]. To test if these molecules are involved in ICH-mediated brain damage, we quantified MPO activity and TNF-α mRNA expression in the injured brain of WT and TLR2 KO mice. At 24 h post-ICH, 510 U/g of MPO activity was detected in the ipsilateral hemispheres of the WT mice, whereas a decrease to 200 U/g was seen in the TLR2 KO mice (Figure 7G). In addition, TNF-α transcripts were upregulated 43-fold in the WT mice upon ICH, while only a 23-fold increase was seen in the TLR2 KO mice (Figure 7H). These data suggest a possibility that attenuated production of ROS and TNF-α in the TLR2 KO mice may have contributed to the reduction of ICH damage in these mice.

### Neutrophil-attracting chemokine and adhesion molecule expression are attenuated in TLR2 KO mice

**Figure 8:**
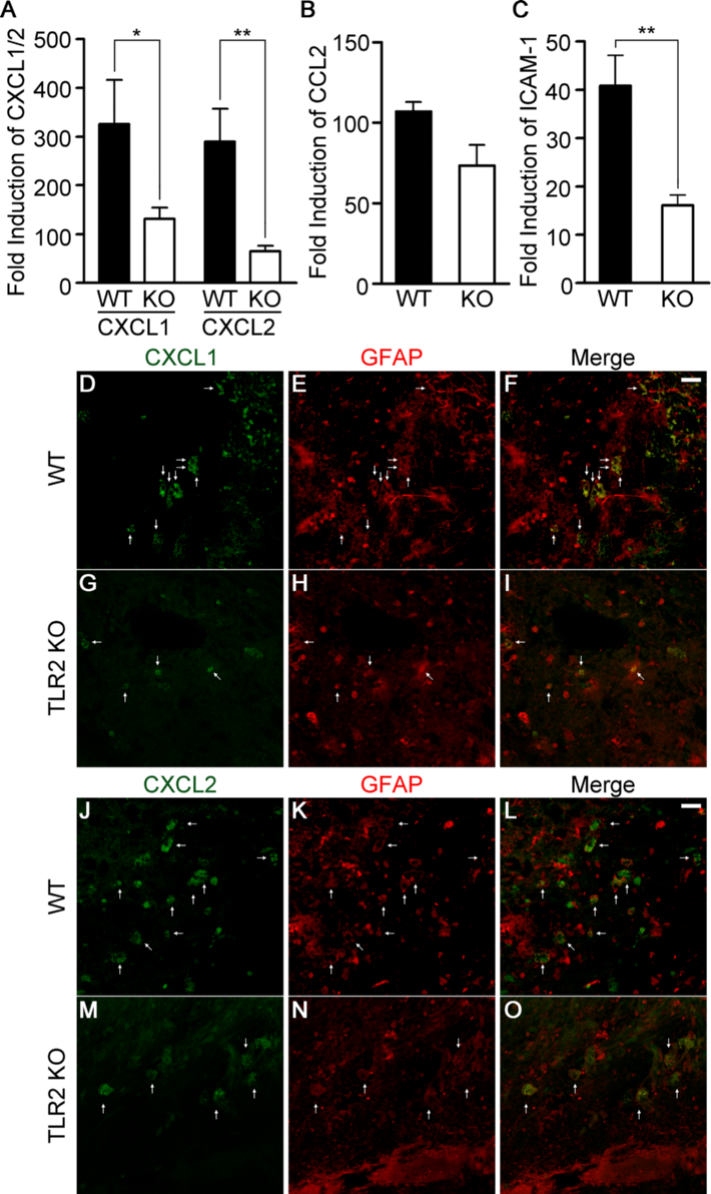
**Neutrophil-attracting chemokine and adhesion molecule expression are attenuated in TLR2 KO mice.** WT and TLR2 KO mice were subjected to collagenase-induced ICH. Six h post-ICH, total RNA was isolated from ipsilateral hemorrhagic tissue (AP, 0.0 to −2.0 mm) and used for quantitative real-time RT-PCR to measure CXCL1 and CXCL2 **(A)**, CCL2 **(B)**, and ICAM-1 **(C)** mRNA levels. Data are expressed as mean ± SEM (** *p* < 0.01, * *p* < 0.05, vs. collagenase-injected WT mice, n = 5). **(D-O)** Brain sections of WT **(D-F,** **J-L)** and TLR2 KO **(G-I,** **M-O)** mice (n = 3) were immunostained with anti-GFAP **(E,** **F,** **H,** **I,** **K,** **L,** **N, and** **O)** and anti-CXCL1 **(D,** **G,** **F, and** **I)** or anti-CXCL2 **(J,** **M,** **L, and** **O)** antibodies 24 h after collagenase injection. Scale bars: 25 μm.

In our flow cytometry experiment, neutrophil infiltration, but not macrophage infiltration, was affected in TLR2 KO mice. This suggests that neutrophil-attracting mechanisms are specifically compromised in the TLR2 KO mice. To address the putative mechanism, the expression of the neutrophil-attracting chemokines CXCL1 and CXCL2 in the injured brain tissue was measured by real-time RT-PCR. The mRNA expression of CXCL1 and CXCL2 was upregulated up to 300-fold in the WT mouse brains at 6 h post-injection (Figure 8A); however, the induction level of CXCL1 and CXCL2 was decreased by 60% and 77%, respectively, in the TLR2 KO mice compared to the WT mice (Figure 8A). The expression of a monocyte-recruiting chemokine (CCL2) was also upregulated (100-fold) in WT mice after ICH (Figure 8B). However, the induction level of CCL2 was not significantly decreased in TLR2 KO mice (Figure 8B). We also tested the expression of ICAM-1, as it has been implicated in the recruitment of neutrophils from the blood. Upon hemorrhagic injury, ICAM-1 transcript was induced 40-fold in WT mice brains, whereas it was upregulated only 13-fold in TLR2 KO mouse brains (Figure 8C). In immunohistochemistry, CXCL1 and CXCL2 expressions were detected primarily in GFAP-IR astrocytes within the peri-hematomal region of WT mice (Figure 8D-F, J-L); however, the expression of both chemokines was significantly attenuated in TLR2 KO mice (Figure 8G-I, M-O). These data suggest that reduced CXCL1/2 and ICAM-1 expression may result in reduced neutrophil infiltration in TLR2 KO mice upon ICH.

## Discussion

In this study, we have shown for the first time that TLR2 plays a detrimental role in brain damage due to ICH, which was tested in two different ICH animal models: collagenase-induced ICH and blood injection-mediated ICH. Reductions in hemorrhagic volume, neurological deficit, BBB compromise, neutrophil infiltration, and inflammatory gene expression were observed in TLR2 KO mice as compared to WT mice. Previous studies using an ischemic brain injury model, such as MCAO, have shown conflicting results as to the role of TLR2. While some studies showed decreased brain injury in TLR2 KO mice after a focal ischemia [23,24], another study showed decreased neurological function and increased infarct size in TLR2 KO mice, suggesting a neuroprotective effect of TLR2 [25]. Our data are in line with the previous reports showing neurotoxic effects of TLR2 in a MCAO stroke model [23,24], yet the mechanisms of the detrimental TLR2 effects in our ICH model are distinct from those in the ischemic brain injury model.

In our study, we found that TLR2 contributes to collagenase-induced BBB damage and MMP9 activation in astrocytes in an ICH model. The critical role of MMP9 in BBB damage has been well documented [10,29]. In the ischemic injury models, MMP9 activity was detected in endothelial cells, astrocytes, and neurons [34,35], and HMGB1 released during stroke was implicated in activation of MMP9 via TLR4 signaling [36,37]. In our study, the ICH-induced MMP9 activation in astrocytes was dependent on TLR2. This implies that certain TLR2 endogenous agonist other than HMGB1 released during ICH would activate MMP9 in astrocytes via TLR2 and thereby exacerbate BBB damage. In addition, in an ischemic injury model, blockade of TLR2 signal by neutralizing antibody blocked macrophage infiltration into the injured brain parenchyma [38], suggesting a critical role in brain damage for TLR2 expressed on macrophages, not brain-resident glia. Furthermore, the absence of TLR2 signaling did not affect neutrophil recruitment to the infarct region in ischemic injury model [23]. However, in the collagenase-induced ICH model, TLR2 expression did not affect macrophage infiltration, rather it regulated neutrophil infiltration. In addition, our data from heterologous blood transfusion experiments show that TLR2 KO mice are less susceptible to the blood transfusion-induced brain damage compared to WT mice, regardless of the genotype of the blood injected. Although these data cannot be exactly extrapolated to the collagenase-induced ICH, these data argue that TLR2 expression in the cells of brain parenchyma may play an important role in the detrimental effects of TLR2. These data suggest that the pathogenic mechanism of neuroinflammation following stroke is quite distinct depending on the type of initial insult (ischemia vs. hemorrhage).

In our study, MMP9 activity was detected in astrocytes, but not in microglia. Still, considering TLR2 expression in microglia, we do not exclude the possibility that TLR2 may also exert its proinflammatory and detrimental effects by activating microglia independently of astrocyte MMP9 activation. It also must be noted that, in our *in vivo* data, MMP9 was also expressed in neurons, yet we could not detect TLR2 expression in neurons (data not shown). It can be conjectured that neuronal MMP9 activation is induced by inflammatory cytokines such as TNF-α that are released during hemorrhage. Indeed, it has been reported that MMP9 can be induced in TNF-α-stimulated neuronal cells [39], and we found that TNF-α was induced in the ICH-induced brain in a TLR2-dependent manner, supporting this possibility.

In our study, we found that neutrophil infiltration after ICH is reduced in TLR2 KO mice. Neutrophil infiltration following ICH may cause tissue damage by generating ROS and secreting proteases [40]. We found strong induction of MPO activity in the ICH-injured WT mice which was ameliorated in the TLR2 KO mice. Oxidative stress by MPO is implicated in neuronal cell death [41-43]. Reduced neutrophil infiltration may therefore account for the attenuation in ICH-induced damage volume observed in TLR2 KO mice. Taken together, our data propose a novel mechanism in secondary hemorrhagic brain injury in which TLR2 activation on perihematomal astrocytes leads to MMP9 activation and BBB disruption, which may augment secondary brain damage following ICH. It implies that the mechanisms of secondary neuroinflammatory responses in hemorrhagic stroke are distinct from those in ischemic stroke, and therefore should be clinically dealt with in different fashions. In conclusion, we demonstrated in this study that TLR2 plays a detrimental role in ICH-induced brain damage, at least partly by activating MMP9 in astrocytes and recruiting neutrophils. This suggests that TLR2 may be a novel therapeutic target for treatment of ICH.

## Conclusions

In this study, we revealed that TLR2 plays a detrimental role in brain injury following ICH in a mouse model. ICH-induced hemorrhagic lesion and neurological deficit were significantly reduced in TLR2 KO mice. Similarly, astrocyte MMP9 activation and subsequent BBB damage after ICH were attenuated in TLR2 KO mice compared to WT mice. The reduced BBB leakage in TLR2 KO mice accompanied with attenuated neutrophil infiltration and proinflammatory/neurotoxic gene expression, which may account for the attenuated ICH damage in these mice. Taken together, our data show that TLR2 contributes to secondary inflammatory brain damage after ICH by activating MMP9 in astrocytes and compromising BBB integrity.

## Materials and methods

### Animals

TLR2 KO mice [44] were generously provided by Dr. S. Akira (Department of Host Defense, Osaka University, Osaka, Japan). The TLR2 KO mice had been backcrossed to the C57BL/6 background for more than 10 generations; C57BL/6 mice purchased from Koatech (Pyeongtaek, Korea) were used as WT control mice. The mice were housed at 23 ± 2°C with a 12 h light–dark cycle and fed food and water *ad libitum*. All surgical and experimental procedures were reviewed and approved by the Institutional Animal Care and Use Committee (IACUC) at Seoul National University.

### ICH model and hemorrhagic injury volume analysis

The procedure for inducing ICH by collagenase injection in mice was carried out as described previously [45]. Briefly, WT and TLR2 KO mice (8–10 week old males, 22–25 g) were anesthetized using sodium pentobarbital (30 mg/kg body weight, i.p.), and placed on a stereotaxic apparatus (myNeuroLab, St. Louis, MO, USA). Animals were injected with PBS or collagenase VII-S (0.075 U in 0.5 μl PBS; Sigma, St. Louis, MO, USA) at a rate of 0.5 μl/min into the right caudate putamen (stereotaxic coordinates in mm with reference to bregma: AP, −1.0; ML, −3.0; DV, −3.5) using a 26 G needle. In the ICH by blood infusion model, mice were injected with blood (20 μl) prepared from the tail vein at a rate of 1.5 μl/min. Five min after the injection finished, the needle was removed in three intermediate steps over 3 min in order to minimize backflow. The incision was cleaned with saline, sutured, and the animals were kept on a warm pad during recovery. To prepare brain tissue sections, animals were deeply anesthetized with sodium pentobarbital and perfused intracardially with saline followed by cold 4% paraformaldehyde (PFA) in a 0.1 M phosphate buffer. The brains were removed, post-fixed overnight in the same fixative at 4°C, rinsed twice with PBS, and placed in 10, 20, and 30% sucrose in PBS, serially, for 48 h at 4°C. The brains were then quickly frozen and cut into serial coronal sections (50 μm thickness) in a cryostat (CM3050S, Leica, Germany). Sections were collected as free-floating sections in cold PBS and then used for histochemical analysis. Five coronal brain slices from different levels of the injured hemorrhagic area were selected from each mouse brain and used for cresyl violet staining. The injury area was quantified using Imagepro Plus software (Media Cybernetics, Inc., Rockville, MD, USA), and the injury volume was calculated in cubic millimeters (mm^3^) by multiplying the section thickness by the measured injury areas as described elsewhere [46]. An experienced experimenter blinded to the mouse genotypes ran the Imagepro Plus software and performed the calculations and analysis.

### Primary glial cell culture

Primary mixed glial cultures were prepared as previously described [47]. Briefly, mixed glial cultures were prepared from postnatal day 1–3 WT or TLR2 KO mice. After removing meninges from the cerebral hemisphere, tissue was dissociated into a single-cell suspension by gentle repetitive pipetting. Cells were cultured in DMEM supplemented with 10 mM HEPES, 10% FBS, 2 mM L-glutamine, and 1X antibiotic/antimycotic in 75 cm^2^ flasks at 37°C in a 5% CO_2_ incubator, and the medium was changed every 5 days. After 2 weeks, microglia were removed from culture by treating with 100 mM L-leucine methyl ester for 60 min, then harvesting by trypsinization (0.25% trypsin, 0.02% EDTA); cells were then seeded in 6-well dishes or poly-D-lysine coated-glass slides, depending on the experiment.

### Evaluation of neurological deficit

Neurological deficits were assessed at 24 and 72 h following collagenase injection. An experimenter blinded to the mouse genotypes scored all mice for neurological deficits using a 28-point neurological scoring system [27]. The tests included body symmetry, gait, climbing, circling behavior, front limb symmetry, and compulsory circling. Each test was graded from 0 to 4, establishing a maximum deficit score of 28. The mice were sacrificed for analysis immediately following testing.

### Determination of BBB permeability

To evaluate BBB permeability, mice were intraperitoneally (i.p.) injected with Evans blue dye (2% in saline, 4 ml/kg) at 2 days after collagenase injection. After 24 h, brain tissues were harvested, fixed with 4% PFA, and placed in 30% sucrose in PBS for 48 h at 4°C. The brains were then quickly frozen and cut into serial coronal sections (50 μm thickness) in a cryostat. A single section was collected out of every 8 consecutive sections, mounted on a slide, and then visualized under a light microscope. The Evans blue dye-stained area in each section was quantified using Imagepro Plus software, summed throughout the injured hemorrhagic brain area, and the total injury volume was calculated in cubic millimeters (mm^3^) by multiplying the section thickness by the measured area.

### MPO activity assay

The extent of neutrophil infiltration was assessed by measuring MPO activity as described previously [48,49]. In brief, mice were sacrificed with an overdose of sodium pentobarbital 24 h after ICH, and transcardially perfused with saline. Brains were extracted and dissected free of the olfactory bulbs and the cerebellum. The ipsilateral hemispheres were weighed and homogenized in a 20 mM phosphate buffer (pH 6.0) containing 0.5% hexadecyltrimethyl ammonium bromide. Following centrifugation at 18,000 xg for 30 min at 4°C, 0.1 ml of supernatant was added to 0.6 ml of 0.1 M phosphate buffer (pH 6.0) with 0.05% H_2_O_2_ containing 2 mg/ml of *o*-dianisidine. After 10 min of incubation the reaction was stopped by adding 0.1 ml of 1% NaN_3_, and the rate of absorbance change was measured at 460 nm in a spectrophotometer (BioRad, Hercules, CA, USA). MPO activity was calculated using a standard curve prepared with purified MPO (Sigma) and expressed as units per gram (U/g) of tissue.

### Real-time RT-PCR

Real-time RT-PCR was performed using SYBR Green PCR Master Mix (ABI, Warrington, UK) as described previously [50]. Reactions were performed in duplicate in a total volume of 10 μl containing 10 pM primer, 4 μl cDNA, and 5 μl SYBR Green PCR Master Mix. The mRNA levels of each target gene were normalized to that of GAPDH mRNA. Fold-induction was calculated using the 2^-∆∆CT^ method, as previously described [51]. All real-time RT-PCR experiments were performed at least three times, and are presented as mean ± SEM unless otherwise noted. The following sequences of primers were used for real-time RT-PCR: TLR2 forward: 5′-CCT AGA AGT GGA AAA GAT GTC GTT CA-3′; TLR2 reverse: 5′-GAA GAA AAC GGA ATT CTC TTT TCG AC-3′; TNF-α forward: 5′-AGC AAA CCA CCA AGT GGA GGA-3′; TNF-α reverse: 5′-GCT GGC ACC ACT AGT TGG TTG T-3′; CXCL1 forward: 5′-CCG AAG TCA TAG CCA CAC TCA A-3′; CXCL1 reverse: 5′-GCA GTC TGT CTT CTT TCT CCG TTA C-3′; CXCL2 forward: 5′-AGA CAG AAG TCA TAG CCA CTC TCA AG-3′; CXCL2 reverse: 5′-CCT CCT TTC CAG GTC AGT TAG C-3′; CCL2 forward: 5′-TCA GCC AGA TGC AGT TAA CG-3′; CCL2 reverse: 5′-GAT CCT CTT GTA GCT CTC CAG C-3′; ICAM-1 forward: 5′-GAT CAC ATT CAC GGT GCT GG-3′; ICAM-1 reverse: 5′-GAG AAA TTG GCT CCG TGG TC-3′; MMP-9 forward: 5′-CAT TCG CGT GGA TAA GGA GT-3′; MMP-9 reverse: 5′-ACC TGG TTC ACC TCA TGG TC-3′; GAPDH forward: 5′-CAC CCT GTT GCT GTA GCC GTA T-3′; GAPDH reverse: 5′-AGG TCA TCC CAG AGC TGA ACG-3′.

### Immunofluorescence staining

Immunostaining was carried out using previously established procedures [50]. The sections were incubated in a blocking solution (5% normal donkey serum, 2% BSA, and 0.1% Triton X-100) for 1 h at room temperature (RT). The sections were then incubated overnight at 4°C with the following antibodies: mouse anti- TLR2 (1:200; eBioscience, San Diego, CA, USA), mouse anti-NeuN (1:2,000; Millipore, Billerica, MA, USA), rabbit anti-GFAP (1:10,000; DAKO, Denmark), rat anti-CD68 (1:1000; AbD Serotec, Raleigh, NC, USA), mouse anti-MMP9 (1:500; abcam, UK), rat anti-Gr-1 (1:1,000; Invitrogen, Carlsbad, CA, USA) goat anti-CXCL1 (1:100; Santa Cruz, Dallas, TX, USA), goat anti-CXCL2 (1:100; Santa Cruz). The sections were incubated for 1 h at RT with FITC- or Cy3-conjugated secondary antibodies (1:200; Jackson ImmunoResearch, West Grove, PA, USA), and then mounted on gelatin-coated slides and coverslipped with VectaShield medium (Vector Labs, Burlingame, CA, USA). Images were captured using confocal laser scanning microscopy (LSM5 PASCAL; Carl Zeiss, Germany).

### Gel zymography

Cells were incubated with or without 2 μg/ml of Pam3 in serum-free media at 37°C in a 5% CO_2_ incubator for 24 h and supernatants were collected. To concentrate protein, 3 volumes of ethanol was added to supernatant, and the mixture was incubated at −20°C for 1 h and centrifuged at 18,000 xg for 30 min at 4°C. Precipitates were resuspended in sample buffer (0.25 M tris pH 6.8, 10% glycerol, 2% SDS) and subjected to SDS-PAGE in 8% polyacrylamide gels containing 1 mg/ml gelatin. After electrophoresis, gels were incubated with 2.5% Triton X-100 for 30 min at RT and incubated with zymography developing buffer for 30 h at 37°C. The gels were stained with 0.25% Coomassie blue and destained to visualize MMP9 activity.

### *In situ* zymography

*In situ* gelatinolytic activity was measured on frozen sections (16 μm thickness) as described previously [30]. At 24 h following ICH, brains were removed and immediately frozen on dry ice. Fresh sections were incubated with fluorescein-conjugated DQ gelatin substrate (Invitrogen) for 2 h and fixed and mounted with VectaShield medium. Cleavage of DQ gelatin by MMPs results in green fluorescent products. After gelatinolytic activity had been assessed, some sections were fixed with 4% PFA and incubated with primary antibodies specific to neurons (NeuN) or astrocytes (GFAP; Millipore), followed by Cy3-conjugated secondary antibodies. Images were then captured using confocal laser scanning microscopy.

### Flow cytometry

To measure inflammatory cell infiltration in the brain, flow cytometry was performed. Single cell suspensions were prepared from ipsilateral hemispheres 72 h after ICH, washed with 2% fetal bovine serum in PBS, and incubated with Fc blocker™ (BD Biosciences, San Jose, CA) for 10 min at 4°C. After washing twice with 2% FBS in PBS, cells were incubated with PE-conjugated CD45, APC-conjugated CD11b, and FITC-conjugated GR-1 antibodies (BD Biosciences) for 30 min at 4°C. A BD FACSCalibur flow cytometer (BD Biosciences) was used to measure microglia (CD45^int^, CD11b^+^, Gr-1^−^), leukocyte (CD45^hi^), macrophage (CD45^hi^, CD11b^+^, Gr-1^−^), and neutrophil (CD45^hi^, CD11b^+^, Gr-1^+^) populations as defined elsewhere [52,53]. Data were collected and analyzed using the BD CellQuest™ System (BD Biosciences).

### Statistical analysis

Statistically significant differences between the WT and TLR2 KO mice were determined by paired Student’s *t*-test or one-way ANOVA with Tukey’s multiple comparison test. All data are presented as the mean ± SEM, and differences were considered significant when the *p* value was less than 0.05.

## Abbreviations

ICH: Intracerebral hemorrhage
BBB: Blood–brain barrier
MMP: Matrix metalloproteinase
TLR: Toll-like receptor
LTA: Lipoteichic acid
HSP: Heat shock protein
CD: Cluster of differentiation
HMGB-1: High mobility group box-1
WT: Wild-type
KO: Knock-out
SCI: Spinal cord injury
GFAP: Glia fibrillary acidic protein
IR: Immunoreactive
NeuN: Neuronal neuclear antigen
Gr-1: Granulocyte differentiation antigen 1
ROS: Reactive oxygen species
MPO: Myeloid peroxidase
TNF-α: Tumor necrosis factor alpha
CXCL: Chemokine (C-X-C motif) ligand
ICAM: Intercellular adhesion molecule
MCAO: Middle cerebral artery occlusion
